# Does quality of life assessment in palliative care look like a complex screening program?

**DOI:** 10.1186/1477-7525-11-7

**Published:** 2013-01-14

**Authors:** Gianluca Catania, Massimo Costantini, Monica Beccaro, Annamaria Bagnasco, Loredana Sasso

**Affiliations:** 1 IRCCS A.O.U. San Martino – IST Istituto Nazionale per la Ricerca sul Cancro, Genoa, Italy; 2 Department of Health Sciences, University of Genoa, Genoa 16132, Italy

**Keywords:** Quality of life, Outcome assessment, Palliative care, Complex intervention

## Abstract

**Background:**

Palliative Care (PC) is an approach that improves the Quality of Life (QoL). A number of QoL assessment tools have been developed and validated in PC. It is not clear how QoL should be measured in PC practice. A procedure of QoL assessment in clinical practice can be defined as a clinical intervention focused on QoL assessment. This is a typical complex intervention that should be appropriately developed and described in all its components and assessed for its effectiveness. The aim of this study is to define a framework to help researchers to develop and evaluate clinical interventions focused on QoL assessment in PC.

**Methods:**

A study group of experts in PC and in research methodology was set up to define a framework that would describe the principles of clinical interventions focused on QoL assessment in PC. The study group discussed the WHO Population Screening Principles as a possible useful framework. The new principles had to be developed taking into account the following criteria: 1) specific to PC practice; 2) address a single underlying characteristic; 3) anchored to relevant literature; 4) consistent with the WHO PC definition.

With regard to contents and the format of the principles, discussions occurred among the study group members through a cognitive process.

**Results:**

We reviewed each of the WHO Population Screening Principles and adapted them to QoL assessment, taking into account the defined criteria. As a result, a new framework, *the QoL Assessment Principles in Palliative Care* was developed. It consisted of 4 sections, for a total of 11 principles.

**Conclusions:**

The WHO Screening Principles framework was used to outline the eleven essential principles to be considered in developing and/or evaluating clinical interventions focused on QoL assessment in PC. *The QoL Assessment Principles in Palliative Care* identified could represent a methodological and ethical standard to be considered when developing and evaluating a clinical intervention focused on QoL assessment in PC.

## Background

### Quality of life in palliative care

Palliative Care is an approach that improves the Quality of Life (QoL) of patients and their families facing the problem associated with life-threatening illness, through the prevention and relief of suffering by means of early identification and impeccable assessment and treatment of pain and other problems, physical, psychosocial and spiritual [1].

According to the World Health Organization (WHO) definition of health, formulated in 1948, in healthcare systems QoL is related to health and its physical and psychosocial dimensions [2]. In palliative care, a theoretical concept with a pragmatic approach is the Calman’s gap theory that described QoL as an individual experience conceptualized as the gap between a person‘s life experience and expectations [3]. QoL is described as a dynamic, subjective and multidimensional concept including physical, emotional, psychological, social and spiritual dimensions [4-6]. Such dimensions affect each other as well as patients’ overall QoL [4].

The challenge of assessing QoL in palliative care must take into account that QoL is based on the perspectives and priorities of the individual patient [7], where all the subjective dimensions interact with each other [4]. In addition, response shift phenomenon needs to be considered when measuring QoL, such phenomenon involves a change in the meaning of patients’ self-evaluation of QoL as a result of a change over time in their internal standards, values and priorities [8].

### Quality of life assessment

A number of QoL assessment tools have been developed and validated in palliative care [9] and their use described for research purposes [10], to support clinical practice [11], and as part of the quality programs [12].

For research purposes, a number of observational studies have described and analysed QoL impairments of palliative care patients in different settings [13]. Moreover, QoL has also been used as an outcome measure in observational [14], quasi-experimental [15] and experimental studies [16]. Two systematic reviews [17,18] concluded that QoL assessment is feasible in clinical trials and has the potential of providing valuable outcomes to further support clinical decision-making.

The European PRISMA group developed a research guidance aimed at improving outcome measures for patients and families [19]. The Institute of Medicine (IOM) identified Comparative Effectiveness Research (CER) in cancer care as a high priority where the ability to measure QoL is central to CER in oncology because survival and disease-free survival do not properly incorporate outcomes that are significant for policy-makers, physicians, and patients [20,21].

QoL assessment in research is supported by detailed study protocols and rigorous guidelines (i.e. Good Clinical Practice) reporting the rationale of QoL assessment, the psychometric characteristics of QoL measures, and the detailed procedures for QoL assessment [22].

Higginson & Carr [23] recommended QoL assessments in palliative care for purposes other than research. As part of the quality programs, QoL assessments can be used as a criterion by which services are evaluated, or aimed at improving assessment skills among healthcare professionals or at ensuring that clinical audit focuses on what is important to patients rather than on the technical aspects of quality.

In support to clinical practice, QoL assessments have been proposed to assess variations in individual patients over the disease trajectory and to ensure that the individual plan of care is centered on the patient rather than on the disease [15,16,23].

### The challenge of quality of life assessment in clinical practice

A procedure of QoL assessment in clinical practice (i.e. a routine procedure and a thorough QoL assessment in palliative care patients) can be defined as a clinical intervention focused on QoL assessment. According to the definition proposed by the Medical Research Council (MRC) [24], it is a typical complex intervention that should be appropriately developed and described in all its components and assessed for its effectiveness [25].

A basic prerequisite for starting a process of assessment is to identify the optimal intervention and analyse all its components, and identify mechanisms that lead to improved outcomes and potential barriers to its application in a specific context [26].

A theoretical framework for the development-evaluation-implementation process of a complex intervention has been developed [24]. This framework has never been applied to a clinical intervention focused on QoL assessment in palliative care, and no consensus exists on what should be the essential components of the intervention. A specific framework for developing a clinical intervention focused on QoL assessment in palliative care could be a useful guideline that would help stakeholders to better define the intervention in all its components and make it suitable for use as a reference either in practice, research or quality programs purposes.

### Aim of the study

The aim of this study is to define a framework to help researchers to develop and evaluate clinical interventions focused on QoL assessment in palliative care.

## Methods

In January 2011, a study group of experts in palliative care and in research methodology (one epidemiologist, one research nurse, one palliative care physician and two university nursing professors) was set up to define a framework that would describe the principles of clinical interventions focused on QoL assessment in palliative care. The study group’s members came from two different institutions in the north of Italy – a teaching and research hospital and a university - and started working together to support one of the authors (GC) in his doctoral program.

As outlined in the latest MRC framework guidelines and from the standardization perspective, we used here the term “component” according to Hawe’s view [27] where the constant aspects of the intervention are the essential functions and the variable aspects are their form tailored to local conditions.

The study group discussed the WHO Population Screening Principles [28] as a possible useful framework, as both interventions share a number of characteristics:

• both are complex interventions with a number of interacting components sensitive to features of local contexts that present specific problems in standardizing and implementing the intervention, and in assessing its effectiveness;

• both interventions are focused on assessment procedures. The screening is focused on diagnostic tests (i.e. smear test) offered to apparently healthy people for the early detection of a disease or condition. The QoL clinical intervention is focused on assessment tools (i.e. the Palliative Outcome Scale – POS [29] offered to patients to identify and prioritise issues in one or more dimensions of QoL;

• the assumption for both of these interventions is that early detection can positively modify, for screening programs, the natural history of the disease and, for QoL clinical interventions can modify the natural history of impairments or problems;

• both deal with specific difficulties in defining, developing, documenting, and replicating the intervention and both require the use of qualitative and quantitative methodologies for their evaluation.

The framework of reference for developing a screening program in all its components is the WHO Population Screening Principles [28] that identifies and describes ten principles that could be used as a guideline to plan a new screening intervention. In breast cancer, for instance, such principles have proved to be effective in detecting early-stage disease and reducing overall mortality [30].

Wilson & Junkner [28] listed the WHO Population Screening Principles at the time when technological advances in medicine made screening a topic of growing importance [31]. Similarly, patients’ QoL is the *core* element of healthcare practice in palliative care, in which QoL assessment has been proposed as the intervention aimed at improving practice related to screening for problems, targeting interventions, and monitoring outcomes in palliative care [23]. It showed a positive effect of QOL data on physician–patient communication specifically focusing on continuity of information, facilitating rapport and inter-personal communication between patients and healthcare professionals [32].

By using the ten WHO Population Screening Principles [28] as a framework, we developed the principles of clinical interventions focused on QoL assessment in palliative care, as specified in the following procedures.

### Procedures

Firstly, the study group members met to sharing the proposal of one of the author (MC) to use the WHO Screening Principles as a framework to develop the principles of clinical interventions focused on QoL assessment in palliative care. We all agreed that the principles had to be developed taking into account the following criteria:

1. specific to the palliative care practice;

2. address a single underlying characteristic;

3. anchored to relevant literature in the Pubmed database using key words and synonymous related to each single principles (e.g. palliative care, disease trajectory, assessment tool);

4. consistent with the WHO palliative care definition.

One of the authors (GC) developed a first draft version of the WHO Screening Principles adapted to QoL assessment. The format and the contents of the principles were discussed and emended during five meetings guided by one of the author (MC) through a cognitive process [33].

Such meetings were aimed at getting to an agreed final version of the principles. Consensus was reached when all the study group’s members agreed a unanimous decision to the final version.

## Results

According to the development stage of the MRC framework for complex interventions [24], we assumed that a clinical intervention focused on QoL assessment should be based on a number of components that form a coherent structure, and that all such components - standardized by form and functions - linked the interventions to the expected outcomes.

In this article, we hypothesized a supporting theory based on the WHO Population Screening Principles [28] that we modified and contextualized to QoL assessment in palliative care practice. The WHO Population Screening Principles [28] describe the principles of a “complex intervention” (i.e. the population screening program) for an early identification of a disease or a pre-disease condition in healthy subjects.

*The QoL Assessment Principles in Palliative Care* consisted of the following 4 sections, for a total of 11 principles (see Table 1): section one includes three principles related to “the problem” (i.e. the QoL impairment); section two includes two principles dealing with the assessment tool; section three includes one principle focusing on the treatment/intervention; and section four includes five principles dealing with “the clinical intervention focused on QoL assessment”.

**Table 1:** Application of WHO screening principles to QoL assessments in palliative care

**THE WHO SCREENING PRINCIPLES**	**THE QoL ASSESSMENT PRINCIPLES IN PALLIATIVE CARE**
**The disease, condition**	**The problem (the QoL impairment)**
1. The condition should be an important health problem, in terms of prevalence (e.g. breast cancer) or for the serious consequences if not early discovered and treated (e.g. phenylketonuria).	1. The problem should be a serious condition for the patient either in terms of prevalence (e.g. pain, depression) and/or distress for the patient (e.g. itch, hiccup) or the result of late detection and management of the problem (e.g. a new or unusual distressing symptom occurred over the disease trajectory).
2. There should be a recognizable latent or early symptomatic stage.	2. The problem should be highly unlikely to be reported by all the patients or recognized by the professional if not actively assessed.
3. The natural history of the condition, including development from latent to declared disease, should be adequately understood.	3. The trajectory of the problem should be sufficiently understood to assure a timely assessment to anticipate and appropriately address the problem.
**The diagnostic test**	**The assessment tool**
1. There should be a suitable test or examination	1. A validated, reliable and sensitive-to-change tool for detecting and measuring the problem should be available.
2. There should be acceptable for the population	2. The tool should be practical, easy to use and questions must not be distressing for the patients.
**The treatment**	**The treatment - intervention**
1. There should be an accepted treatment for patients with recognized disease.	1. There should be an appropriate treatment/intervention for patients with the recognized problem.
**The screening programme**	**The clinical intervention focused on QoL assessment**
1. There should be an agreed policy on whom to treat as patients	1. There should be an agreed policy on which a problem (or a problem with a certain degree of impairment) has to be addressed with appropriate treatment or intervention.
2. Facilities for diagnosis and treatment should be available.	2. There should be the possibility to appropriately administer the tool, including professionals trained with the procedure.
	3. The treatment-intervention for patients with QoL impairments should be available, including professionals trained for the treatment-intervention.
3. The cost of case-finding (including diagnosis and treatment of patient diagnosed) should be economically balanced in relation to possible expenditure on medical care as a whole	4. The cost of problem-finding (including all the steps from the administration of the tool until the end of the treatment – intervention delivered) should be economically justified.
4. Case-finding should be a continuing process and not a “once and for all” project.	5. QoL assessment should be a continuing process and not a “once and for all” project.

The original WHO Population Screening Principles [28] are reported in Table 1 and, hereafter, *the QoL Assessment Principles in Palliative Care* are italicised and described.

### The Problem (the QoL impairment) (3 principles)

1. The problem should be a serious condition for the patient either in terms of prevalence (e.g. pain, depression) and/or distress for the patient (e.g. itch, hiccup) or the result of late detection and management of the problem (e.g. a new or unusual distressing symptom occurred over the disease trajectory).

The assumption underlying this principle is that screening and monitoring for patients’ problems allow these to be noticed by the healthcare professionals [19]. There is some evidence that when problems are identified, as well as those not necessarily obvious, some or all of them could be addressed by palliative care staff contributing significantly to the improvement of the patients’ QoL [34].

2. The problem should be highly unlikely to be reported by all the patients or recognized by the professional if not actively assessed.

This principle is met because using QoL assessment to identify which patients are mostly at risk of developing impairments in QoL dimensions that are primary in palliative care.

Especially in the case of needs that are either not regularly assessed by staff or not reported by all the patients including spiritual, psychosocial and physical problems such as loneliness, anxiety, dry mouth and pain. As reported by Lunder et al. [35] and LeMay et al. [36] when patients’ needs are not addressed patients may consider ending their lives prematurely.

3. The trajectory of the problem should be sufficiently understood to assure a timely assessment to anticipate and appropriately address the problem.

This principle suggests the need to adequately understand the trajectory of patient-centered outcomes before implementing clinical interventions focused on QoL assessment.

It is crucial to palliative care recognize the trajectory of the problems influencing the QoL dimensions in palliative care patients [37]. For example, being aware of the changing prevalence and severity of the physical, psychological, social, and spiritual needs over the illness trajectory [38,39] is necessary to choose the most appropriate self-report measures of a screening programme and to prevent and address such impaired conditions.

### The assessment tool (2 principles)

1. A validated, reliable and sensitive-to-change tool for detecting and measuring the problem should be available.

This principle suggests the need to measure QoL with instruments that can be comfortably used in clinical palliative care practice and that are valid, reliable and have the characteristic to detect over time any changes in the dimension(s) being measured (i.e. responsiveness). Although there is no agreement on how QoL should be measured [9] a number of literature reviews identified a number of QoL instruments as appropriate for use in palliative care [9,40,41]. For example, the Palliative care Outcome Scale (POS) [29] is one of the most comprehensive, valid, and reliable QoL instruments applied in palliative care patients with a variety of diagnoses – dementia, chronic heart failure, chronic kidney disease, COPD. It showed a statistically significant responsiveness to change between scores on admission and over time [34].

2. The tool should be practical, easy to use and questions must not be distressing for the patients.

This principle underscores the importance of having QoL measures that are acceptable for the palliative care population. Acceptability means that the instrument has to be practical, easy to use and not distressful for palliative care patients, especially when the disease is at a very advanced stage . For example, the Palliative care Outcome Scale (POS) [29] was considered by patients as a valuable and not burdensome communication tool that helped them to identify their individual needs [42].

### The treatment/intervention (1 principle)

1. There should be an appropriate treatment/intervention for patients with the recognized problem.

This principle is particularly important when developing and implementing a clinical intervention focused on the assessment of QoL in palliative care settings, because it highlights which criteria such intervention should meet, the availability of appropriate treatments, interventions and/or services to treat the recognized problem that is the impaired QoL. For example, an impaired physical dimension is commonly treated with an effective medical treatment and despite symptoms are clustered they are controlled without inducing unacceptable side effects [43]. Conversely, looking at spirituality, although it is recognized as an essential dimension of quality by the National Consensus Project for Quality Palliative Care [44] and the National Quality Forum (NQF), there is little evidence on what kind of spiritual care interventions effectively address spiritual needs while delivering palliative care [45].

Such principle focuses primarily on clarifying aspects of palliative care teams decision making that should be evidence based by integrating “the best research evidence with clinical expertise and patient values” [46].

### The clinical intervention focused on QoL assessment (5 principles)

1. There should be an agreed policy in relation to which a problem (or a problem with a certain degree of impairment) has to be addressed with appropriate treatment or intervention.

This principle underlines the importance of defining individualized care plans based on impaired QoL dimensions where interventions are proposed to patients and their families according to the QoL assessment. Impaired conditions should be discussed with patients and a careful evaluation of the causes should be included and followed up. As a result of poor QoL scores or when conditions are worse compared to earlier assessments, policies related to dimensions included in the QoL assessment should be considered to ensure special attention and an appropriate treatment. As part of a larger clinical intervention aimed at fostering the screening of impaired conditions where early detection and ongoing assessment would make a difference in the intervention and outcome, QoL measurement should trigger a process within policies defining what to do and how (i.e. intervention, treatment, professionals trained in delivering the intervention, service to which the patient refers) according to the patients’ care expectations.

2. There should be the possibility to appropriately administer the tool, including professionals trained with the procedure.

This principle indicates that palliative care patients should be assessed for their needs. As palliative care focuses on improving patients’ QoL, resources and facilities (i.e. treatments and services) should be available where patients can find skilled and trained healthcare professionals able to screen and monitor QoL over time.

3. The treatment-intervention for patients with QoL impairments should be available, including professionals trained for the treatment-intervention.

This principle states that patients requiring treatment should be able to obtain it [28] from trained professionals who have the skills to answer to patients needs with appropriate interventions.

4. The cost of problem-finding (including all the steps from the administration of the tool until the end of the treatment – intervention delivered) should be economically justified.

This principle suggests that according to the Agency for Healthcare Research and Quality (AHRQ) [47] a cost-effectiveness analysis of interventions is essential. Similarly, a clinical intervention focused on QoL assessment to establish whether the improvement in patients outcomes justified the expenditures relative to other choices should be considered. As an example, costs could be related to staff time to (1) develop the clinical intervention focused on QoL assessment; (2) administer the QoL measure; (3) train the staff to use the tool; (4) help patients and their families to understand and monitor QoL changes [48].

Furthermore, in palliative care the decision to implement this type of clinical intervention should not be made on the basis of costs alone, but also considering the ethical issues. Researchers have shown that cost savings are accrued when clinical decision making recognizes patients’ preferences, values and needs. From an ethical perspective this means that when treatments that no longer produce a benefit for the patient are discontinued, they can improve the patients’ QoL [49].

5. QoL assessment should be a continuing process and not a “once and for all” project.

This principle addresses the issue that QoL assessment should be made at the baseline and then monitored over time. This approach allows to understand how symptoms and suffering develop, and fluctuate during the illness trajectory [50] and recognize patients’ experiences and evaluate the effectiveness of the interventions [51].

## Discussion

Palliative care aims to help patients and their families have a better QoL by preventing and treating symptoms related to the disease and to the treatment itself, and addressing patients’ psychosocial and spiritual needs [1]. The QoL assessment in clinical practice is an important part of palliative care resulting in several advantages for patients in terms of: 1) problem identification [37]; 2) facilitating patient decision making; 3) monitoring for changes [23],and 4) facilitating communication [32].

Although many QoL measures have been developed specifically for palliative care [9], evidence about effective interventions focused on QoL assessment implemented in clinical practice is lacking [52].

Before undertaking a study for evaluating how an intervention focused on QoL assessment works in everyday practice, it is essential to define the intervention and its components.

In this article, we defined a clinical intervention focused on QoL assessment as a complex intervention because its components interact with one other in a system that, by its very nature, is complex. There is a need to identify and then include the key components of the clinical intervention in an coherent evidence-based structure. Since clinical interventions focused on QoL assessment and the screening programs shared a number of characteristics, the WHO Screening Principles framework [28] was used to outline the eleven essential principles to be considered in developing and/or evaluating clinical interventions focused on QoL assessment in palliative care. Moreover, the principles identified could represent a methodological and ethical standard to be considered when developing and evaluating a clinical intervention focused on QoL assessment in palliative care.

The QoL Assessment Principles in Palliative Care could be a methodological standard because they intended to provide a guidance aimed at developing an organized integrated clinical intervention where all the components along the QoL assessment are planned and interrelated. A single underlying characteristic of the principles was developed in the attempt to provide a detailed guidance to evaluate each component of the clinical interventions focused on QoL assessment. The QoL Assessment Principles in Palliative Care here proposed could also improve clinical practice by considering QoL assessment as a complex intervention and not simply a task where the introduction of the tool in clinical practice is the only important element.

The principles defining the QoL Assessment Principles in Palliative Care may be considered as an ethical standard because they are provided in a way that respects people’s rights with palliative care needs. These principles could ensure that palliative care patients have recognized their right to have poor QoL identified early and followed-up, thus permitting to define appropriate interventions that would maximize benefits and minimize harms and positively influence the quality of patient care delivery and outcomes.

We started developing the QoL Assessment Principles in Palliative Care in a way that has not been previously considered, by identifying both the theoretical basis and the key components using the MRC framework approach. Although the MRC framework approach here applied gives strength to this development stage and to the next steps, the paucity of strong evidence in including each component in the QoL Assessment Principles in Palliative Care may represent a limit for this first stage, as well as that fact that it has been based only on reflection and expertise of this study group.

## Conclusion

We reckon we have found some good claims and a new point of view when looking at QoL assessment in palliative care. Our findings may contribute to answering the initial question we included in the title of this paper and they could be the starting point for further research aimed at defining a framework to develop and evaluate clinical interventions focused on QoL assessment in palliative care.

Although this study provides a new insight on clinical interventions focused on QoL assessment in palliative care practice, our findings are based on intuitions, reflections and experiences of the study group members. Further studies are needed to identify the extent to which a broader group of palliative care experts agree on appropriateness and completeness of the principles described in the QoL Assessment Principles in Palliative Care. Thus, these proposed principles could benefit from a consultation through a Delphi process approach for reaching a wider consensus.

## Abbreviations

QoL: Quality of life; WHO: World health organization; IOM: Institute of medicine; CER: Comparative effectiveness research; MRC: Medical research council; POS: Palliative outcome scale; COPD: Chronic obstructive pulmonary disease; NCP: National consensus project; NQF: National quality forum; AHRQ: Agency for healthcare research quality.

## Competing interests

The five authors have no conflicts of interest, including specific financial interests and relationships and affiliation relevant to the subject matter or materials discussed in the manuscript.

## Authors’ contributions

GC, MC and MB contributed to conception and design of the study. All the authors of the study discussed the theoretical model and results presented in this article. This paper was primarily written by GC, MC and MB and then revised, discussed, and amended by all the authors that approved the final version of the manuscript.
